# Association of Systemic Lupus Erythematosus Clinical Features with European Population Genetic Substructure

**DOI:** 10.1371/journal.pone.0029033

**Published:** 2011-12-14

**Authors:** Elisa Alonso-Perez, Marian Suarez-Gestal, Manuel Calaza, Torsten Witte, Chryssa Papasteriades, Maurizio Marchini, Sergio Migliaresi, Attila Kovacs, Josep Ordi-Ros, Marc Bijl, Maria Jose Santos, Sarka Ruzickova, Rudolf Pullmann, Patricia Carreira, Fotini N. Skopouli, Sandra D'Alfonso, Gian Domenico Sebastiani, Ana Suarez, Francisco J. Blanco, Juan J. Gomez-Reino, Antonio Gonzalez, and for the European Consortium of SLE DNA Collections

**Affiliations:** 1 Laboratorio de Investigacion, Instituto de Investigacion Sanitaria, Universitario de Santiago Hospital Clinico Santiago de Compostela, Santiago de Compostela, Spain; 2 Division of Clinical Immunology and Rheumatology, Department of Internal Medicine, Hannover Medical School, Hannover, Germany; 3 Department of Histocompatibility and Immunology, Evangelismos Hospital, Athens, Greece; 4 Clinical Immunology, Fondazione Istituto di Ricovero e Cura Carattere Scientifico (IRCCS) Ospedale Maggiore Policlinico, University of Milan, Milan, Italy; 5 Rheumatology Unit, Second University of Naples, Naples, Italy; 6 Department of Rheumatology, Hospital of Hungarian Railways, Szolnok, Hungary; 7 Internal Medicine Research Laboratory in Autoimmune Diseases, Hospital Vall d'Hebron, Barcelona, Spain; 8 Department of Rheumatology and Clinical Immunology, University Medical Center Groningen, Groningen, The Netherlands; 9 Rheumatology Department, Hospital Garcia de Orta and Rheumatology Reseach Unit, Instituto de Medicina Molecular, Lisboa, Portugal; 10 Institute of Biotechnology, Academy of Sciences of the Czech Republic, Prague, Czech Republic; 11 Institute of Clinical Biochemistry, Martin Faculty Hospital, Jessenius Medical Faculty, Martin, Slovakia; 12 Rheumatology Department, Hospital 12 de Octubre, Madrid, Spain; 13 Pathophysiology Department, Athens University Medical School, Athens, Greece; 14 Department Medical Sciences and Interdisciplinary Research Center of Autoimmune Disease (IRCAD), Eastern Piedmont University, Novara, Italy; 15 Ospedale S. Camillo - Forlanini, Unità Operativa Complessa di Reumatologia, Roma, Italy; 16 Department of Functional Biology, University of Oviedo, Oviedo, Spain; 17 Laboratorio de Investigación Osteoarticular y del Envejecimiento, Servicio de Reumatología, Complejo Hospitalario Universitario A Coruña, A Coruña, Spain; 18 Department of Medicine, University of Santiago de Compostela, Santiago de Compostela, Spain; Institut Jacques Monod, France

## Abstract

Systemic Lupus Erythematosus (SLE) is an autoimmune disease with a very varied spectrum of clinical manifestations that could be partly determined by genetic factors. We aimed to determine the relationship between prevalence of 11 clinical features and age of disease onset with European population genetic substructure. Data from 1413 patients of European ancestry recruited in nine countries was tested for association with genotypes of top ancestry informative markers. This analysis was done with logistic regression between phenotypes and genotypes or principal components extracted from them. We used a genetic additive model and adjusted for gender and disease duration. Three clinical features showed association with ancestry informative markers: autoantibody production defined as immunologic disorder (*P* = 6.8×10^−4^), oral ulcers (*P* = 6.9×10^−4^) and photosensitivity (*P* = 0.002). Immunologic disorder was associated with genotypes more common in Southern European ancestries, whereas the opposite trend was observed for photosensitivity. Oral ulcers were specifically more common in patients of Spanish and Portuguese self-reported ancestry. These results should be taken into account in future research and suggest new hypotheses and possible underlying mechanisms to be investigated. A first hypothesis linking photosensitivity with variation in skin pigmentation is suggested.

## Introduction

Systemic Lupus Erythematosus is an autoimmune disease with a very varied spectrum of clinical manifestations [1]. It can affect multiple tissues and organs including kidneys, joints, skin, pleura and pericardium, diverse blood cells and the nervous system. It is also associated with a large variety of auto-antibodies and abnormalities of the immune system. These features are not present in all patients or at all times in the same patient. The disease course alternates flares and periods of remission and clinical presentation can be different in subsequent flares from the observed previously in the same patient. This clinical heterogeneity poses many challenges to clinical diagnosis, treatment and research. Unfortunately, our understanding of its causes is still very incomplete, although it seems that genetic, environmental and socioeconomic factors have a role.

Recent Genome Wide Association studies (GWAS) have provided a list of more than 30 confirmed SLE susceptibility loci [2]. Some of them have been associated with particular SLE clinical features, but they are far to explain its clinical heterogeneity [3]. Other studies have pointed to a broad effect of genetics in the form of the specific genetic background of human subpopulations. There has been knowledge of differences in SLE phenotype between continental ethnic groups for decades [1], [4], but only research in recent years has been able to confirm the importance of genetic background by discriminating between genetics and socioeconomic or environmental factors [5], [6], [7]. The demonstration of an effect of genetic background in SLE phenotype provides the foundation for exploring the possibility that substructure within an ethnic group could influence also the disease clinical presentation. Recent work in about 1900 European-American SLE patients seems to support this hypothesis by showing correlation between the prevalence of some clinical features and ancestry informative markers (AIMs) [8], [9]. These markers are SNPs that had shown in previous studies large differences in allele frequency between Europeans from different ancestries [10], [11], [12], [13]. The finding of these correlations between clinical features and European substructure is very important to discriminate between the different factors influencing SLE heterogeneity and it is possible it could increase our power to identify etiological relationship for the different SLE phenotypes.

Our aim has been to explore the influence of European population substructure in the SLE phenotype of about 1400 European SLE patients from 9 countries. Three of the 12 clinical features analyzed, production of autoantibodies, oral ulcers and photosensitivity, were associated with informative European AIMs confirming the likely effect of variation in genetic background within the European ethnicity.

## Materials and Methods

### Ethic statement

All patients gave their written informed consent to participate and sample collection and study was approved by the relevant ethics committees at each of the recruiting centres. The project was approved by the Comite de Investigacion Clinica de Galicia (Spain).

### Patient data

**Figure 1:**
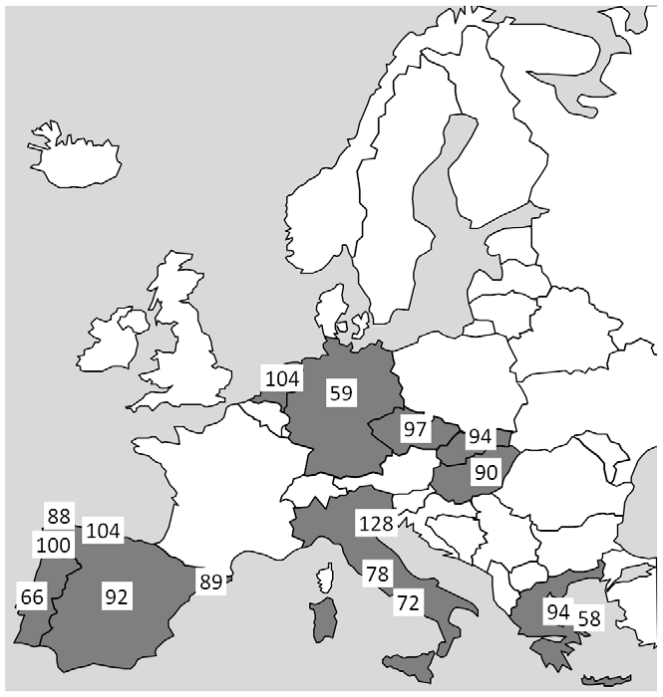
Collections of SLE patients with number of patients available for analysis.

Samples from 1413 European SLE patients recruited at 16 centres from nine different countries were collected as described [14]. Patients were questioned about their ancestry and only patients with uniform ancestry from the country of origin were included. Data retrieved from each patient included the 11 SLE American College of Rheumatology (ACR) classification criteria [15], age of disease onset, disease duration and gender (**Table 1**). Immunologic disorder was defined as for these critera and included mainly anti-dsDNA or anti-Sm positive patients. Antinuclear antibodies (ANA) were not included in the analysis because they were almost uniformly present in all patients. Each recruiting centre contributed a mean of 88.3 SLE patients with range from 58 to 128 patients (**Figure 1**).

**Table 1:** Clinical characteristics of the patients with SLE.

Characteristic^a^	% (95% C.I.)	mean ± S.D.
Women	89.5 (87.9–91.1)	
Age of onset		31.1±13.1
Disease duration		11.9±8.3
Malar rash	55.7 (53.0–58.4)	
Discoid rash	17.8 (15.8–19.8)	
Photosensitivity	52.4 (49.7–55.1)	
Oral ulcers	28.0 (25.6–30.4)	
Arthritis	80.3 (78.2–82.4)	
Serositis	35.5 (33.0–38.0)	
Renal disorder	40.5 (37.9–43.1)	
Neurologic disorder	13.6 (11.8–15.5)	
Hematological disorder	71.3 (68.9–73.7)	
Immunological disorder^b^	78.7 (76.5–80.9)	
ANA	91.4 (89.8–93.0)	

a Data from >98% of the patients for all characteristics except for the following: malar rash, neurologic disorder, hematologic disorders and age of disease onset with data from >92% of the patients; disease duration and ANAs that were not available from two recruiting centres (available in >80%).

b Defined as for the SLE ACR classification criteria [15] including abnormal anti native DNA, anti-Sm antibodies, LE cells or false positive serologic syphilis test.

### Genotyping of AIMs

Six AIMs were determined in the SLE patients: rs6730157, rs382259, rs4988235, rs12203592, rs354690 and rs12913832. The three first are the most informative AIMs in differentiating Northern from Southern European subpopulations identified in a study analyzing 300 000 SNPs in 4000 European subjects [11]. Results from rs4988235 were not used for analysis because it was largely redundant with rs6730157 in our samples (r^2^ = 0.87). rs12203592, rs354690 are the two AIMs more informative for East-West place of origin inside Europe according to the same study [11]. rs12913832 is a SNP associated with large differences in frequency across Europe and unrelated with the previous [10]. These 5 SNPs were amplified in a single PCR reaction done with the KAPA2G fast HotStart (Kapa Biosystems, Woburn MA, USA) on a final volume of 10 µl ( 20 ng genomic DNA) , using 3 mM MgCl2 and 0.2 µM of each primer. Products were purified by Exo-SAP digestion with Exonuclease I (Epicentre, Madison, WI) and Shrimp Alkaline Phophatase (GE Healthcare, Barcelona, Spain). Subsequently, single-base extension reactions with the SNaPshot Multiplex kit (Applied Biosystems, Foster City, CA) were done. Samples were analyzed in an AbiPrism 3130xl Genetic Analyzer (Applied Biosystems). Genotyping call rate success was 99.7%. Sequences of primers and probes are available from the authors upon request.

### Statistical analysis

We have computed the allelic frequencies of the AIMs per each of the 16 recruiting centres to assess their variability and whether they follow the previously reported trends along Europe. Concordance of these genotypes with Hardy-Weinberg equilibrium (HWE) was also assessed by each of the centres given that one of the causes of deviation is population stratification and the AIMs have population specific frequencies. The *P* value for claiming deviation from HWE was set at 0.01, a conservative threshold taking into account the number of centres and AIMs. Factor analysis via principal component (PC) extraction was applied to the AIM genotypes to reduce dimensionality. Association of each of the ACR classification criteria with each of the AIMs and with the retained principal components was analyzed by logistic regression. Genotypes were coded according to an additive model (0, 1 and 2, for the common homozygote, the heterozygote and the rare homozygote genotypes, respectively). The results that are presented included gender and disease duration as covariates. The odds ratio by each allele (O.R.) and their 95% confidence intervals (C.I.) are also given. Analysis of association of age of disease onset with the AIMs genotypes or with the PC was done with multiple linear regression. Genotypes were coded in a similar way, and gender was included as a covariate in these analyses. Analyses were also conducted with inclusion of the recruitment centre as covariate and without any covariates and if results changed interpretation, this circumstance was reported. All statistical analyses were done with Statistica 7.0 (StatSoft, Inc., Tulsa, OA). A significance threshold of 0.008 was applied according to a Bonferroni correction for the six AIMs analyzed.

## Results

### AIM genotypes and population substructure

None of the five AIMs was significantly deviated from HWE in any of the sample collections (*P*>0.01). They showed a large variation between patients with different self-reported ancestry within Europe (**Table 2**). The most extreme difference was observed for rs6730157 that showed an A allelic frequency of 15.5% in Greek patients, and of 73.6% in Dutch patients. The most restricted range of frequencies was observed for rs354690 (from 37.1% to 45.9% frequency of the T allele). Three of the AIMs, rs6730157, rs12913832 and rs382259, showed a clear differentiation between patients from Southern European countries (Portugal, Spain, Italy and Greece) and those from Central Europe (The Netherlands, Germany, Czech Republic, Slovakia and Hungary). This is in agreement with major axis of known European population substructure [11], [12], [13].

**Table 2:** Allele frequency of the ancestry informative markers (AIMs) by country of self-reported ancestry.

Country^a^	rs6730157 A	rs12913832 G	rs382259 T	rs12203592 T	rs354690 T
Greece	0.155	0.367	0.439	0.037	0.452
Italy	0.160	0.417	0.485	0.113	0.435
Portugal	0.386	0.258	0.689	0.075	0.409
Slovakia	0.403	0.699	0.688	0.102	0.441
Hungary	0.426	0.670	0.646	0.084	0.371
Spain	0.430	0.329	0.678	0.145	0.407
Czech R.	0.479	0.792	0.768	0.149	0.459
Germany	0.578	0.767	0.698	0.121	0.397
Netherlands	0.736	0.808	0.798	0.067	0.4237

a Countries are ordered according to allele frequencies of rs6730157. The SNPs are from most variable to less variable, left to right.

We applied factor analysis to the genotypes of the five AIMs and two PC explaining 46.5% of the variance were retained. Rs6730157 was the main contributor to PC1, whereas rs12203592 was the main contributor to PC2. These PC showed significant correlations with the geographical coordinates of the patient's ancestries (**Table 3**): PC1 with the latitude (r = −0.47) but not with the longitude; and PC2 correlated with both the latitude (r = −0.13) and the longitude (r = 0.10). Some of the individual AIMs showed a stronger correlation with these coordinates than others and the correlations were particularly strong with latitude (Table 3).

**Table 3:** Correlation between the two first principal components (PC) obtained from the AIM genotypes and between each of the AIMS with the geographic coordinates of the reported ancestries of the SLE patients.

	Latitude		Longitude	
PC or AIM	r	*P*	r	*P*
PC1	0.47	<10^−6^	0.03	ns
PC2	−0.13	10^−6^	0.10	1.4×10^−4^
rs6730157	0.71	0.002	−0.40	ns
rs382259	−0.62	0.010	0.50	0.048
rs12913832	0.91	10^−6^	0.42	ns
rs12203592	0.20	ns	−0.58	0.019
rs354690	−0.07	ns	0.41	ns

### Association of SLE clinical features with population substructure

**Figure 2:**
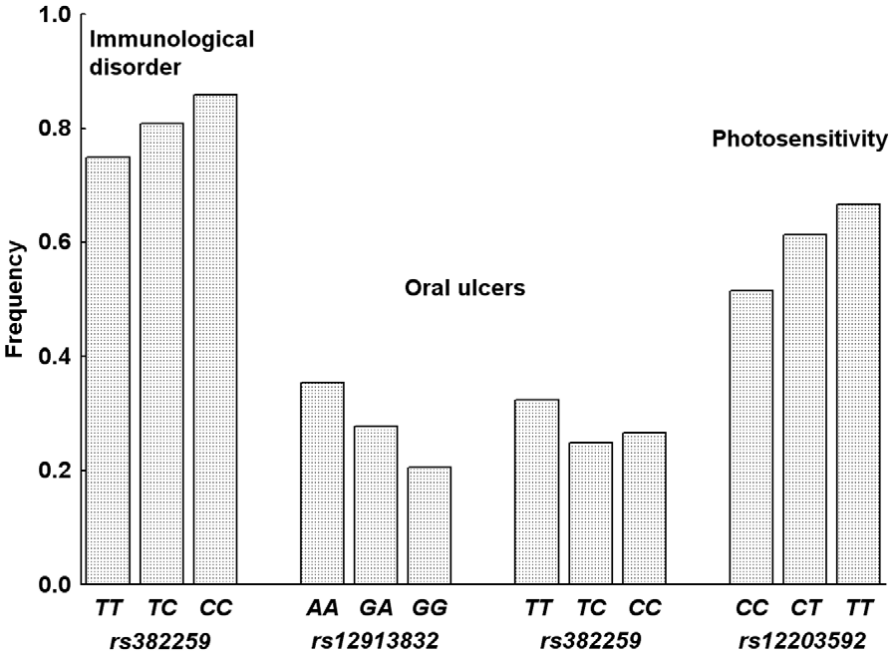
Frequency of the SLE clinical features associated with AIM genotypes. The abscise axis indicates the AIM genotype and in the ordinate axis are the frequencies of the indicated clinical manifestation.

Once we had confirmed that the five AIMs were informative for European population substructure in our patients, we used them to look for evidence of its effects in the phenotype of SLE. Three of the ACR classification criteria showed association with some AIMs. The presence of the immunological disorder criterion, which consists in production of a variety of specific autoantibodies (mainly anti-dsDNA or anti-Sm antibodies), was associated with rs382259 (*P* = 6.8×10^−4^; O.R. = 0.70, 95% C.I. = 0.57–0.86). Increased prevalence of immunological disorder was associated with the CC and TC genotypes of rs3822259 (**Figure 2**). Association was also found between immunological disorder and PC1 (*P* = 0.004; O.R. = 0.82, 95% C.I. = 0.72–0.94).

Oral ulcers were also associated with two AIMS: rs12913832 (*P* = 6.9×10^−4^; O.R. = 0.73, 95% C.I. = 0.61–0.87) and rs382259 (*P* = 9.3×10^−4^; O.R. = 0.72, 95% C.I. = 0.59–0.87) in single AIM analyses. Association with these two AIMs persisted in multivariate analysis that included the five AIMs (*P* = 2.0×10^−4^ and *P* = 1.7×10^−3^, respectively) indicating that each of the two AIMs have an independent contribution to the association. Higher prevalence of oral ulcers was associated with the AA and GA genotypes of rs12913832, and with the TT but not with the TC genotypes of rs382259 (**Figure 2**). No association with the PCs was observed. Analysis including the recruiting centers as covariates showed that association with the two AIMs was completely dependent on this factor. This was due to the higher frequency of oral ulcers in patients from Spain and Portugal according to their self-reported ancestry than in patients from other origins (47.1% versus 18.5%; P<10^−6^).

Finally, photosensitivity was associated with rs12203592 (*P* = 0.0021; O.R. = 1.47, 95% C.I. = 1.15–1.88). This association was independent of conditional analysis with the other four AIMs (*P* = 0.001). Photosensitivity was more common in patients that were TT or CT for rs12203592 or (**Figure 2**).

The remaining SLE ACR criteria and the age of disease onset were not associated with any AIM or PC.

## Discussion

Our results have confirmed a significant effect of European population substructure on the SLE phenotype. The substructure associated phenotypes, immunological disorder, oral ulcers and photosensitivity, have already been identified in the only other group of SLE patients in which this possibility has been tested [8], [9]. The consistency of results adds credibility to the findings. However, we have not replicated association of other clinical features from the previous study and the effect on oral ulcers was not fully convincing, either in our study or in the previous one [8], [9].

The AIMs we have used were able to show European population substructure. Their variation was mainly marked by the North-South differentiation that has been found in previous studies [10], [11], [12], [13]. This is remarkable because our analysis included only a fraction of the many European populations used in these studies. Of potential relevance is the lack in our study of Scandinavian subjects or of subjects from the British Islands or from Russia that had been included in the studies for the discovery of the AIMs [11], [12], [13]. These populations that correspond to geographical extremes to the North, West and East of the European population could enlarge the range of AIM frequencies and improve correlation of these frequencies with geographical coordinates.

A limitation of the AIMs we have used is that they are not enough for classification of individual subjects. However, we think they were sufficient to detect a large fraction of the SLE clinical features associated with European population substructure. This conclusion is based in two pieces of evidence. The first is that the three findings of our study were associated with more than one AIM indicating a certain level of redundancy in spite of the low correlation between the AIM genotypes (mean pairwise r^2^ = 0.007). The second is that only the very top AIMs contributing to the first two PCs for population substructure in Europeans, according to a study with 300 000 SNPs [11], were informative in our study: the third AIM contributing to PC1, rs4988235, showed a high correlation with the first AIM, rs6730157 (r^2^ = 0.87), and the same pattern of associations (not shown); and rs354690 that was the second AIM for PC2 did not show association with any of the clinical features.

Association of the production of the SLE specific autoantibodies that are included in the immunologic disorder criterion (in most cases, antibodies to dsDNA or the nuclear Sm antigen) with rs382259 and with PC1 indicates a role of the patient's genetic background. It is worth to mention that immunological disorder was also associated with the two first PCs in the study done in European American SLE patients [9]. This association was interpreted as meaning that a Southern and Western European ancestry predisposes to autoantibody production. Our results can be interpreted also as meaning that a Southern European ancestry predisposes to this phenotype, but no clear differentiation in the West-East axis was observed.

The effect of genetic background in the prevalence of autoantibodies had already been shown in relation with the continental ethnic groups. For example, there are reports showing an increased prevalence of anti-Sm and anti-RNP antibodies in African patients relative to Europeans [16], [17], [18], [19]; other differences concern clusters of autoantibodies that include anti-Sm or anti-dsDNA [20], or other specific SLE autoantibodies like anti-P [21] or anti-RNA helicase A [18], [22]. But, there is not any report of a general higher prevalence of SLE autoantibodies in patients of a specific ethnic group. On the contrary, some autoantibodies have been found at low prevalence while others show high prevalence in the same ethnicity [18], [20], [22]. Therefore, it seems likely that genetic background is influencing specific responses more than the general abnormalities leading to antibody mediated autoimmunity. This could be the case for HLA alleles whose frequency is highly variable between populations and that affect prevalence of anti-P [21], anti-cardiolipin and anti-beta2GPI antibodies in different ethnicities [23]. It seems likely that differences within Europeans affect autoantibody production in SLE patients in the same way.

The association of oral ulcers with European population substructure is more open to question. Although we have observed a clear association with two AIMs, rs12913832 and rs382259, it disappeared after adjusting by center of recruitment. This result invites to caution but does not invalidate interpretation because centers of recruitment were strongly linked with the patient's reported ancestries. In fact, classifying the patients by their reported ancestries showed a clear excess of oral ulcers in patients from Spain and Portugal. This increased prevalence is reflected by with the AIMs association with a higher frequency of the associated genotypes in the South-Western European ancestries. Results from the study on European American patients showed an increase of oral ulcers also in patients with Southern European ancestry, but again it did not persist after adjusting for covariates [9]. Given these results, we cannot conclude at present. We cannot distinguish a genuine difference in the phenotype of patients with a South-Western European ancestry from confounding factors associated with recruiting hospitals. In addition, there has not been a wide interest in the analysis of variation in prevalence of oral ulcers between SLE patients from different ethnicities. Large differences between SLE patient series from different ethnicities have been reported [4], but as they were not obtained in comparative studies and have not been replicated, it is unclear whether these differences could be attributable to genetic background. Therefore, we lack evidence of reproducible trends and of possible etiologic factors that could help us to interpret the current results. However, there are data from large collections of European SLE patients that support the difference we have found: two collections of Spanish SLE patients showed a prevalence of oral ulcers of 46.4% and 54.3% (of 462 and 490 patients, respectively) [24], and a study of 544 Portuguese SLE patients reported a prevalence of 45% [25]; whereas the Euro-Lupus study showed a prevalence of 12.5% (of 1000 patients from all over Europe) [26].

The third SLE clinical feature we have found associated with European population substructure is photosensitivity. It was associated with the genotypes of two AIMs. rs12913832 directionwas characteristic of Northern Europeans. An excess of photosensitivity in SLE patients with Northern European ancestry was also found in the previous study of European-Americans [8], [9]. This effect of population substructure is the most amenable to interpretation because it could be related with lighter skin pigmentation, which has a key role in sensitivity to sunburns, melanoma and other UV-related cancers and which is much more common in Northern Europeans than in Southern Europeans. This interpretation is in agreement with the lower prevalence of SLE photosensitivity among African American patients with SLE [19], or black patients from South Africa [27] or Jamaica [28]. However, rs12203592 was also associated with photosensitivity without any discernible geographical frequency distribution, and no studies of skin pigmentation or phototype in relation with SLE photosensitivity have been done in Europeans. Therefore, this hypothesis requires specific testing. It could be done directly by comparing prevalence of photosensitivity in function of skin color and sun exposure, but also by looking for association between the wide array of loci already known to determine skin pigmentation, which show wide differences in frequency among Europeans [10], [29], and SLE photosensitivity.

Other SLE clinical features showed association with European population substructure in the Richman et al. study [9], but not in our study and, therefore, they remain unconfirmed. These include discoid rash, renal disorder, serositis, neurological disorder and malar rash. Some of them were weakly associated in the Richman study, like malar rash and neurological disorder, but the others showed *P* values below 0.01. These differences could be due to lack of power of our study, false positive findings in the Richman study or to differences between SLE patients and study design of the two reports. For example, our study included a high fraction of subject with self-reported ancestry from Southern European countries followed by Central European countries, whereas the European American patients were very markedly of Northern and Western European ancestry [8].

As has been commented already, the clinical features associated with European populations substructure could be due to their relation with SLE loci that show a frequency gradient within Europe. Apart from the HLA alleles [30], we know already of other differences in SLE associated loci between Europeans from different ancestries. The SLE risk allele of PTPN22 R620W shows a higher frequency in subjects from the North and West of Europe than in those from the South [31]. Our Consortium has also shown that a difference in frequencies, although more complex, is present for PD1.3, a SLE risk polymorphism the PDCD1 locus [32], [33]. The A allele is more common in SLE patients than in controls from the North Center of Europe, similar in patients and controls from the Southeast, and more common in controls than in SLE patients from the Southwest of Europe [33]. Notably, the gradient in frequency has been observed only in controls, not in patients. It is possible than these and similar variations in risk allele frequencies explain the associations between SLE clinical features and European population substructure.

One of the limitations of our study is that it does not include a representative sample of SLE patients from all the countries with European ancestry. It will be also desirable to include in the analysis other important characteristics of SLE besides the ACR classification criteria like additional clinical manifestations or any of the damage or disease activity indexes. It will be also beneficial to count with more detailed information about autoantibodies or severity of the different clinical features. A concerted effort will be necessary to obtain this type of high quality data from a large number of patients of multiple European ancestries.

We have not corrected the *P* values for number clinical features analyzed. This was motivated by the difficulty in defining an appropriate level of correction. Two issues make this difficult: the correlation between clinical features that will lead to overcorrection if a Bonferroni approach is used; and the contentious issue of whether association of each clinical feature should be considered as an independent hypothesis or as a unique hypothesis [34], [35].

In summary, our study reinforces the evidence of a significant but modest degree of variation in the SLE phenotype in relation with European population substructure. The differences we have found will help to understand the underlying mechanisms as clues of possible association with particular loci and skin pigmentation are already suggested. Elucidation of these mechanisms will advance our ability to cope with SLE clinical heterogeneity. In addition, this finding is important for the design of SLE clinical projects including patients of European ancestry that until now have been taken as a unit of study or comparison. Accounting for European substructure will be even more important for research aiming to define the relationships between SLE phenotype and genotype. Specifically, our study contributes also to consolidate the association between Southern European ancestry and more prevalence of autoantibody production and less photosensitivity in SLE patients and suggests the possibility of an increased frequency of oral ulcers in patients with South-Western European ancestry.
